# Play robots to develop competences

**DOI:** 10.3389/frobt.2025.1646523

**Published:** 2025-10-13

**Authors:** Erica Panelli, Lorenzo Guerrieri, Andrea Bonarini

**Affiliations:** AI and Robotics Laboratory, Department of Electronics Information and Bioengineering, Politecnico di Milano, Milan, Italy

**Keywords:** play, game, social robot, robot design, disability, competence, skill

## Abstract

Play is a fundamental activity through which humans and animals acquire skills and competencies. Robots are increasingly capable of engaging in playful interactions with humans, offering new opportunities for learning, development, and social connection. Unlike traditional toys, robots possess autonomy and expressive capabilities, enabling them to propose actions, respond meaningfully, and exhibit intentions and emotions. This transforms the nature of play, making it more interactive and adaptive. For individuals with cognitive or physical impairments, robots can serve as predictable and engaging companions that attract attention, foster motivation, and facilitate social interaction in group settings. In this paper, we present a comprehensive framework to support the design of play-oriented robots and activities. Drawing on more than 20 years of research and development, we provide examples of low-cost robotic systems tailored for diverse user needs, including both typically developing individuals and those with disabilities. Through selected case studies, we demonstrate the effectiveness and versatility of our approach in supporting the design and analysis of playful experiences that are inclusive, goal-oriented, and developmentally beneficial.

## 1 Introduction

Play is a fundamental mechanism through which both humans and animals acquire physical, cognitive, and social skills (Piaget, 1976; Sicart, 2014). It is also recognized as a fundamental right by the United Nations General Assembly (United Nations General Assembly, 1989). In educational contexts, play has traditionally been employed to foster the development of skills and competencies, as it combines the pleasure of engaging activities with the opportunity to confront challenges or simply to explore. This occurs within a framework distinct from everyday life, making play a safe, effective and engaging learning tool (Bonarini and Besio, 2022; Bulgarelli and Bianquin, 2017).

Everyone enjoys playing, as it fulfills two fundamental survival needs: the need to understand and anticipate what may happen—-curiosity—-and the need to gain control over situations, ensuring safety and success (Gopnik, 2020; Bonarini and Besio, 2022). In recent years, gamification—-the application of game elements to traditionally non-play activities—-has expanded significantly across various domains. Play has long been an integral part of children’s development also in educational settings. It is incorporated not only during formal teaching periods to support learning but also during recess, which often plays a crucial role in fostering physical, cognitive, and social development.

Typically, people engage in play either without the use of objects or with toys, passive items that take on assigned roles within the play activity. Robots introduce a new dimension to play: they are objects with autonomous behavior. Unlike traditional toys, robots cannot be manipulated entirely at will; instead, they require the player to establish a relationship with an entity that exhibits its own autonomy and a sense of animacy.

The design of play activities involving robots is often guided by the intuition and craftsmanship of individual designers and teachers, with little support from established methodologies. In this paper, we propose a framework for characterizing robot-mediated play activities, in order to support the development of both robotic systems and play experiences with clearly defined objectives and structured guidelines for achieving them.

In the sections that follow, we begin by introducing definitions of play and related constructs to establish the context of our work. We then present a framework that centers on the characteristics of the intended outcomes of robot-mediated play activities. Finally, for each element of the framework, we provide illustrative examples of robots and play activities, accompanied by a description of the observed effects in its application.

## 2 Play

The term “play” encompasses a wide range of interpretations and meanings across disciplines and cultures (Eberle, 2014; Ruckenstein, 1991; Caillois, 1967; Garvey, 1990).

In line with the position adopted by the EU COST Action “LUDI: play for children with disabilities^1^” we refer to the definition of play proposed by Garvey: “Play is a range of voluntary, intrinsically motivated activities associated with recreational pleasure and enjoyment” (Garvey, 1990). This definition is broad and flexible at the same time, includes all possible types of ludic activities, and considers three significant dimensions, typical of play: it is pleasant, voluntary, and intrinsically motivated (Bulgarelli and Bianquin, 2017).

Play-like activities that are imposed or externally directed are not considered “play for the sake of play”, which is the focus of our research. Only genuine play—-self-directed and intrinsically motivated—-can lead players into the optimal psychological state of flow (Csikszentmihályi, 1997), a condition that fosters personal growth and development.

We also adopt the classification of ludic activities synthesized by the LUDI network from foundational literature (Piaget, 1976; Vygotskij, 1987). This classification distinguishes two main dimensions of play: a *cognitive* dimension, which includes practical play, symbolic play, constructive play, and play with rules (i.e., games); and a *social* dimension, which comprises solitary, parallel, associative, and cooperative play (Besio et al., 2017b; Bulgarelli and Bianquin, 2017). It is important to note that games represent just one specific form of play, characterized by explicit rules that are understood and accepted by all participants. In other forms of play, goals and rules may also be implicit, self-imposed, or evolve during the activity.

These definitions apply to all individuals, including children with disabilities, whose physical, cognitive, and social development can naturally benefit from play activities while experiencing enjoyment.

Every year, millions of toys that can be classified as robots (International Standards Organization, 2012) enter the market. These products typically fall into three main categories: (1) robots that respond to simple stimuli (e.g., reacting when a toy “food” item is brought to their mouth), (2) robots that require basic programming by the user, and (3) robots that exhibit simple, pre-defined behaviors when directly controlled via joysticks or buttons. While these toys can offer brief moments of engagement, this paper focuses on more complex robotic play activities designed to sustain interaction over longer periods and foster deeper cognitive, emotional, and social involvement.

In many rehabilitation or treatment contexts, particularly for individuals with neuro-developmental disorders, robots are frequently integrated into play activities (Cabibihan et al., 2013; Kozima et al., 2009; Robins et al., 2012; Robins and Dautenhahn, 2014; Saleh et al., 2021). However, these activities often do not fully meet the definition of play as outlined in this paper. Instead, they may be more accurately described as play-like rehabilitation interventions (Bonarini and Besio, 2022), where the primary aim is therapeutic rather than intrinsic enjoyment or voluntary engagement.

In the literature, the emphasis is often placed on the design of robot-assisted play activities for children with ASD, with a primary focus on the interaction process. The features of the robots themselves are usually taken as given, either because commercially available platforms are employed or because custom robots are designed within the constraints of existing technical competences.

A widely adopted approach begins with the identification of specific developmental objectives, such as fostering joint attention, turn-taking, imitation, or emotion recognition (Diehl et al., 2012; Scassellati et al., 2012). These objectives are then mapped to play domains, as in the IROMEC framework (Robins et al., 2010), which distinguishes sensory, communication, motor, cognitive, and social-emotional areas. Building on this foundation, the design of a play activity is typically structured as a six-step process, outlined below

1. *Define developmental objectives*—establish the targeted skills or capacities to be fostered in children with ASD, including preferred play formats (van Straten et al., 2020).
2. *Select the relevant play domain(s)* — situate the objectives within one or more of the above-mentioned categories.
3. *Identify activity goals*—determine the specific outcomes the activity should achieve within the selected domain(s).
4. *Design interaction scenarios*—specify the sequence of actions, roles, and interactions between children and the robot (Marti et al., 2009).
5. *Implement activity mechanics*—define the concrete tasks, stimuli, and robot behaviors that will operationalize the scenarios. It is important to match the child’s sensory and cognitive profile, maintaining low sensory load and providing consistent contingencies (Pennazio, 2017). Include positive reinforcement and adjustable difficulty. Organize play into short, repeatable loops (cue → child action → feedback → celebration) to sustain engagement and support frequent success (Santos and et al., 2023).
6. *Facilitation and scaffolding*—assign explicit roles to adults or peers to model behaviors, prompt participation, and guide the generalization of skills to human–human play (Begum et al., 2016).
7. *Evaluate and refine*—assess the activity in practice, gathering feedback to adapt and improve its effectiveness. Collect data linked to the targeted outcomes, and include transfer measures to evaluate whether learned behaviors appear without the robot (Diehl et al., 2012).

Following these steps, adapted from clinical and design-oriented guidelines, robot-assisted play activities can be expected to be purposeful, engaging, and aligned with developmental goals, while remaining adaptable to the needs of individual children.

It should be noted that these guidelines emphasize the personalization of activities to the needs of a single child with autism. In contrast, the design of group activities involving children with diverse needs, including typically developing peers, requires attention to more general principles of play design that aim to engage all participants while still accounting for individual differences. Moreover, existing guidelines are often oriented toward play-like activities proposed by caregivers within therapeutic contexts, rather than toward the development of genuine play opportunities in which children can freely participate (Besio et al., 2017a).

The use of a simple robot in emergent play activities with young children has been explored by Samuelsson (2023). However, in this study, the robot was mostly treated as a conventional toy, with limited attention given to the potential for co-designing the robot and the play activity. Moreover, most of the observed play scenarios did not fully leverage the robot’s interactive capabilities.

A more structured analysis of robots available at that time for use in playful rehabilitation was conducted in Cook et al. (2010), where key characteristics were identified in relation to their potential for supporting play. In our work, we aim to highlight similar and additional aspects in greater detail, with the goal of putting in evidence the realistic possibilities to support not only the choice but also the design of robots for play activities.

The IROMEC project (Ferrari et al., 2009; Robins et al., 2012) represented a notable effort in the direction of developing a set of basic play scenarios along with a robot specifically designed to engage children with autism in therapeutic play. However, only one robot was developed and the range of different potentialities was not explored, thus not exploited.

## 3 Robot features

Our contribution centers on defining the key characteristics of play and robots that can guide the design and implementation of play activities in which robots take a significant role. We propose a framework aimed at achieving developmental outcomes across physical, perceptive, cognitive, and social domains to support the integrated development of robots and play activities. In this section, we examine the characteristics of robots that are relevant for play, either to assess their presence in existing commercial platforms that might be adopted, or to inform the design of new robots that exploit them. The emphasis is placed on how these features can affect the implementation and success of play activities.

Robots are physical entities capable of autonomous movement and interaction with the external world (International Standards Organization, 2012). According to this definition, different types of robots can be designed or selected to match the requirements of a specific play activity.

**FIGURE 1:**
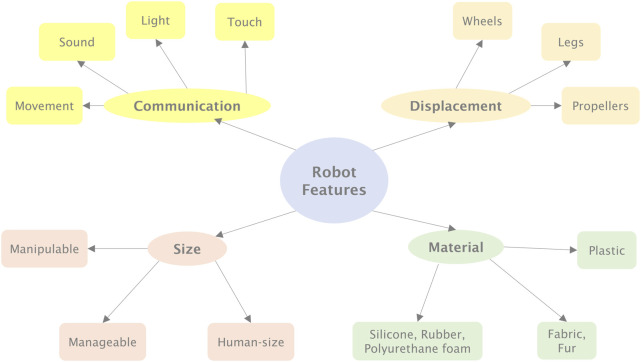
The robot features considered in the design of play activities with robots.

As a first step, our framework identifies the key features that characterize a robot and examines their potential impact on the play experience, as summarized in Figure 1.

### 3.1 Size

We consider three main categories of robot size, defined according to the interaction possibilities they afford.

- Manipulable robots: in the range of 25 cm in height. These robots can be held in the hands, easily picked up, lifted, or even thrown.
- Manageable robots: in the range of 50 cm in height. While not designed for full manipulation, the possibility to keep control over them can be easily perceived, as they remain smaller than children.
- *Human-sized robots*: up to 120 cm in height. These robots can either engage children on a one-to-one scale or create an up-down interaction depending on the child’s size.

### 3.2 Material

The material covering the external surface of a robot influences both the tactile interaction and the imaginary associations it evokes. Different materials can suggest different roles or personalities and affect how the robot is inviting to be touched. Some common options include.

- *Plastic*: A rigid and durable material, typically non-deformable and not particularly pleasant to touch. It often conveys a mechanical or technological impression.
- *Fabric and fur*: Often used over a soft underlying structure, these materials are generally pleasant to touch and familiar to children through plush toys, evoking warmth and emotional comfort.
- *Rubber, polyurethane foam, or silicone*: Softer than plastic and commonly used as a covering for rigid structures, these materials can offer tactile elasticity and are suitable for features like soft limbs or protrusions.

### 3.3 Displacement and movement

Robots can either remain stationary or move through space using various modes of locomotion which may enable play activities that dynamically exploit spatial relationships, such as following, searching, or chasing.

- *Wheels*: Suitable for movement—-also at high speed—-on relatively flat terrain. In indoor environments, omnidirectional wheels can be employed to enable smooth, natural, and unconstrained motion, closely approximating the flexibility of human locomotion.
- *Legs*: Realistic legged locomotion remains challenging, with limited success in commercially available robots. Small humanoids and animal-like robots are still moving very slowly, bigger ones have not an affordable price for the play market. A notable exception is seen in small, vibrating-legged robots, such as toothbrush-like bug robots.
- *Propellers*: These mechanisms enable three-dimensional movement. However, when employed in quadrotors or drones, they are often perceived as potentially hazardous. In contrast, when used to make balloon-based robots float, they are generally regarded as safer and more child-friendly.

Beyond locomotion, other parts of the robot’s body, such as the head or arms, may also move. These movements can produce expressive gestures or be functionally integrated into play actions. Care has to be taken when integrating them in a robot body, e.g., by connecting them to the body through elastic joints, since they offer affordance to be strapped.

Among movement characteristics, both *speed* and *acceleration* play significant roles in shaping the play experience. For example, if children are expected to compete with the robot in speed-based activities, the robot should be able to safely move at a speed of at least 3 m/s. Additionally, expressive gestures, such as trembling or sudden motions to convey emotions like anger or fear, require high acceleration to appear lifelike and believable.

### 3.4 Communication

A robot is expected to engage in some form of communication to be perceived as a genuine play companion. Communication can take place through different channels, each varying in cognitive and perceptual demands.

- *Movement*: Gestures are a fundamental component of human communication, used to convey both semantic content and emotional states. In robots, gestures can be performed through whole-body movements or using specific parts such as the head, limbs, or other appendages, if present. Conversely, perceiving and responding to gestures, facial expressions, or complex movements often requires advanced AI and may depend on costly or cloud-based computation. However, simpler signals, such as distance, relative speed, touch, color, sound intensity, or frequency, can be processed with low computational demands and can play a significant role in shaping the interaction during play.
- *Sound*: Auditory communication encompasses non-verbal sounds (e.g., tones, beeps), musical elements (e.g., jingles), and verbal language, which may be pre-recorded or dynamically synthesized. While modern AI enables real-time speech generation, privacy and consistency concerns can arise with cloud-based solutions, and real-time onboard processing may still be resource-intensive and costly. In general, the production of sound should also take into account the environmental context. While sounds can be effective also with a low quality, verbal messages must be delivered with sufficient clarity and volume to be perceived by the player within the specific setting. If understanding the verbal content is critical to the activity, it should be presented in a form and at a pace that matches the cognitive and perceptual abilities of the player. Ideally, the design of the play activity should ensure that failure to understand a message does not result in a deadlock or halt the interaction. Additionally, all auditory signals should be designed to serve a clear functional purpose. For example, rewarding or pleasant sounds should not be associated with undesirable actions, as this could inadvertently reinforce behaviors that conflicts with the intended goals of the game.
- *Light*: Visual communication through light can range from simple colored LEDs to sophisticated displays. These may present static or animated images (such as eyes or facial expressions) or even textual content, when the player can read it. Also for this channel, we have to guarantee that the visual signal can be perceived in the environmental conditions.
- *Touch*: Touch is typically used to receive input from the player. This input can come from simple buttons, either hidden within the robot’s body or visibly presented, from capacitive touch sensors (including touch screens), or from pressure-sensitive sensors capable of distinguishing different levels of pressure that can be used to classify the type of touch. Touch signals can be employed to respond to specific requests or to play a central role in the activity itself, as in the case of a tag game.

## 4 Player’s abilities

**FIGURE 2:**
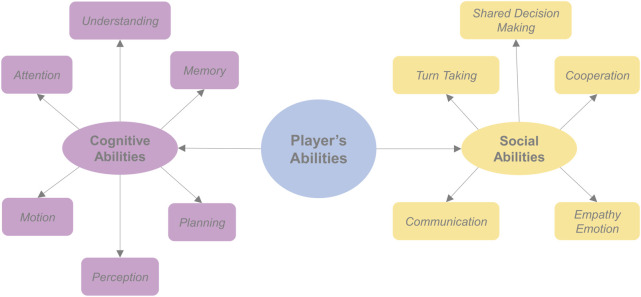
The player’s abilities considered in the design of play activities with robots.

In this section we discuss about cognitive and social abilities of the player, summarized in Figure 2, to be considered when defining play activities.

### 4.1 Cognitive abilities

Cognitive abilities encompass a wide range of functions that may vary depending on an individual’s developmental stage, which is influenced by age and possible conditions such as ADHD, ASD, or other neuro-developmental disorders. Here, we focus on the most commonly addressed cognitive skills in the context of play activities.

#### 4.1.1 Attention

Attention refers to the subject’s ability to focus on an object or activity over time. To support attention, it must first be activated and then sustained through engaging stimuli. As discussed earlier, curiosity and the desire to master a challenge are two primary drivers that help maintain engagement. Curiosity can be triggered by novelty, which may stem from the robot’s unusual or particularly appealing shape, either unfamiliar enough to provoke interest, or familiar enough to evoke positive associations, for instance with well-known cartoon characters. Similarly, the robot’s behavior-expressed through its movements, sounds, and lights-can contribute to capturing attention.

Once the subject is attracted, it is essential to maintain engagement by introducing new stimuli that renew curiosity or by prompting actions that require active participation in the game.

#### 4.1.2 Understanding

Understanding involves constructing a model of an observed phenomenon. In the context of rule-based play, this means comprehending and adhering to the rules in order to participate meaningfully—effectively entering into a sort of “contract” to follow them. Accordingly, a robot involved in such activities should be capable of following the rules, and ideally, of recognizing whether other players are doing so as well. In less structured play, understanding the robot’s behavior contributes to its perceived believability, which often relies on the behavior being clearly goal-directed and interpretable. 1n some cases, even deceptive behavior by the robot can be engaging and valid, provided it aligns with the characteristics of the play activity (de Oliveira et al., 2021). Conversely, behavior that appears random may be difficult to interpret and thus disengaging, while overly predictable or repetitive behavior can quickly lose its appeal.

#### 4.1.3 Memory

Memory plays a central role in many play activities, supporting the ability to retain and recall information, rules, sequences, or associations over time. Both long-term and short-term memory can be stimulated in robot play. Short term memory may be needed to remind sequences of actions that have to be performed to obtain answers from the robot, and may see the active participation of the robot in a sort of turn-taking play. Longer term memory may be needed to remember the rules of the game, or a robot behavior seen some time ago, or even in previous play sections. To trigger this, the signs for that behavior should be interesting enough to provide a sort of imprinting, such as a nice jingle, or an unexpected behavior. The level of complexity should be carefully adapted to the child’s abilities, increasing gradually to promote learning without inducing frustration.

#### 4.1.4 Planning

Planning refers to the capacity to analyze a situation, identify a goal, and determine steps to reach it. This is a complex activity, which requires both attention, understanding and memory. Robots can stimulate this ability through play scenarios involving challenges or puzzles. A planning activity may be induced by having the robot reacting to some stimuli (e.g., different sounds, the positioning of objects, a specific touch action, …) that the player can produce in sequence to make the robot reaching, for instance, a specific position. Such tasks can foster logical reasoning, planning, and cause-effect understanding. The level of challenge should be dynamically adjustable based on the player’s responses, keeping the activity within the optimal range of difficulty to sustain engagement and growth.

#### 4.1.5 Perceptual abilities

Perception is a complex construct that maps sensor signals to models to be used as interface to the real world. Perceptual abilities involve processing and interpreting sensory information such as visual, auditory, and tactile stimuli. Robots offer a unique opportunity to engage these senses in different ways. Visual perception can be stimulated through light patterns, facial expressions, or moving parts; auditory perception through varied sound cues; and tactile perception through different materials and textures on the robot’s surface or the recognition of different types of touch, for instance hugs, pats, punches, caresses. Synchronous multi-modal signals are effective only if all channels convey coherent signals, reinforcing the message rather than overloading the child,as, for instance, when an emotional movement is accompanied by a corresponding sound. Activities might involve identifying the source or direction of a sound, matching colors or shapes, or obtain a response from tactile feedback. These tasks can be especially useful for children with sensory integration challenges, allowing gradual exposure in a controlled and playful context. When designing play activities for all, we have to consider possible specificity of the players. For instance, children with ASD may be overwhelmed by unfiltered stimuli, or disturbed by too strong ones, such as loud sounds. Children with inability to distinguish some colors cannot play in activities where colors are involved.

#### 4.1.6 Motor abilities

Motor abilities include both gross motor skills (e.g., walking, running, jumping) and fine motor skills (e.g., grasping, pointing, manipulating objects, touching in specific ways). Robots can encourage physical activity through movement-based games, such as following the robot, avoiding it, or pressing buttons. Smaller, manipulable robots can support fine motor development through actions like grasping, or positioning. Coordination is mainly related to the proper control of muscles, and can be stimulated by including in the play activity the need of coordinated movements such as sequences of movements, touches, or gestures, and actuation of quick and challenging sequences. For children with physical disabilities, robots can be adapted to accommodate alternative forms of interaction, such as using special interfaces or gesture recognition. Designing play activities that involve movement not only supports motor development but also promotes overall engagement and physical wellbeing.

### 4.2 Social abilities

Social abilities pertain to how individuals interact with others during play. These interactions can involve different configurations—such as peer-to-peer, child-adult, or child-robot relationships—as well as various modes of engagement, including cooperative or parallel play (see Section 2). Notably, a robot may function as an autonomous player or as an avatar controlled by a peer or an adult, allowing the human operator to participate in the game with a distinct role or enhanced abilities.

#### 4.2.1 Turn-taking

Turn-taking is a foundational element of social play, and learning to manage turns appropriately can be challenging. While most robots lack the ability to distinguish between players or to reliably assess turn compliance, human peers or adult facilitators can help structure turn-taking dynamics. A robot can support this process by explicitly calling on individual players, prompting them to act in turn, and thereby reinforcing the concept of turn-based interaction. In any way, the robot helps anchor the game structure while leaving key social roles to human participants.

#### 4.2.2 Cooperation

Cooperative play involves pursuing a shared goal that requires coordinated actions by multiple participants. Robots can facilitate cooperation by acting as interactive objects that provide real-time feedback based on player collaboration. For instance, a robot designed to avoid nearby obstacles might require a group of players to coordinate their movements to steer it toward a target. Success in such a task depends on the group’s ability to work together effectively, thereby fostering essential cooperative skills.

#### 4.2.3 Shared decision making

Play scenarios involving robots can also support the development of shared decision-making abilities. When a group of players must choose how to interact with a robot, they are prompted to negotiate, deliberate, and agree on a plan. Although the robot itself does not take part in the discussion, it can embody the outcomes of decisions through its actions. These scenarios create opportunities for players to experience group dynamics, explore differences of opinion, and develop strategies that are transferable to real-world social situations.

#### 4.2.4 Communication

Communication is the means by which content is shared with others, and it plays a vital role in interactive play. In the context of robots, communication presents specific challenges due to current technological limitations. Despite rapid advancements, robots are still unable to engage in natural, flexible dialogue—especially in dynamic and informal settings like play—without relying on cloud-based resources. This reliance raises concerns regarding privacy and reliability, particularly when interacting with fragile users such as children.

To avoid confusion or mistrust, robot roles in play activities should not rely on open-ended verbal dialogue, which can pose technical challenges due to limited computational resources or network connectivity, as well as ethical concerns over sensitive content. Communication can instead be conveyed through gestures, sounds, music, or carefully curated pre-recorded speech. Non-verbal sounds and music are generally well-received by children (as shown by research demonstrating their role in fostering social bonding with caregivers and supporting early language development, (Pino et al., 2023; Savage et al., 2021)) and naturally complement a moving agent, enhancing expressiveness and perceived animacy when aligned with physical movements.

All communication signals should be clear and effective within the activity context. Written text on screens is often unsuitable, as it requires extra cognitive effort in fast-paced games and in general the ability to read from a suitable support.

On the other hand, it is natural for individuals—-especially children—-to attempt to communicate with a robot using familiar human modalities such as speech, eye gaze, and gestures. However, most robots are not equipped to accurately perceive or interpret these nuanced signals. This mismatch can lead to frustration or confusion if not addressed in the design phase. To mitigate this, play activities should be structured in ways that do not depend on high-level communication channels. Instead, designers can prioritize more accessible and reliably interpretable forms of interaction—such as touch, proximity, or simple button presses—to convey intent and facilitate engagement. These modalities are not only easier for robots to detect and respond to, but also reduce cognitive load for the user, making the interaction more fluid and enjoyable.

#### 4.2.5 Empathy and emotion

Emotions play an important role in human play, affecting motivation, engagement, and social bonding. When designing play activities involving robots, it is important to consider how emotional experiences can be elicited and expressed. While robots are not capable of truly experiencing or recognizing emotions, they can be designed to simulate emotional expressions through integrated multi-modal cues including movement, sound, and light. For example, a robot may “tremble” to suggest fear, emit joyful sounds while moving fast and changing often the direction of movement to express excitement, or use light rhythm to signal emotional states such as anger or calmness synchronized with movement and possibly sound.

The emotional responses of players can also be influenced by the robot’s behavior. A robot that reacts contingently to player actions—-such as responding with a “happy” gesture and music when a goal is achieved—-can foster a sense of empathy. Emotional content is especially important in inclusive play, where fostering a safe, enjoyable, and engaging environment is critical to support all players, including those with disabilities.

However, care must be taken to ensure that emotional cues are unambiguous and appropriately matched to the context of the game. Overly complex or misleading emotional behaviors may lead to confusion or discomfort. Emotional expression in robots should be designed to be simple, consistent, and supportive of the overall play experience.

## 5 General characteristics of play activities

Once the characteristics of robots and players involved in play have been established, we can examine how to leverage these features to design inclusive play activities that target specific developmental abilities. In this section, we introduce general considerations for designing play activities, while the following section presents a set of concrete play activities along with the associated experiences.

### 5.1 Safety

Until recently, robots typically operated in restricted spaces inaccessible to people. With the rise of social robots, exoskeletons, and home robots, the concept of safety has evolved. Robots intended for play should be intrinsically safe, meaning their mass, speed, acceleration, shape, and, if necessary, behavior are designed to ensure that proper use cannot cause harm (Feil-Seifer and Matarić, 2011).

However, intrinsic safety alone is not enough in play contexts: players must also perceive the interaction as safe (perceived safety) (Bartneck et al., 2009; Rubagotti et al., 2022). For example, when playing with a drone with propellers rotating at 10,000 rpm, protective guards are necessary, but the robot should also demonstrate that it will not come closer than a distance considered as safe, say 2 m. Similarly, a fast robot used in a chase game should clearly signal that it cannot harm children, for instance through a soft body and a soft protective safety belt.

### 5.2 Accessibility

Play activities should be accessible to all participants, including children with disabilities. All signals required for the activity must be reliably perceivable and usable by both the robot and the players. For example, if sound or light signals are essential to the game, the environment should support their perception—avoiding excessively noisy spaces, low lighting, or strong sunlight. Accessibility is important when children with disabilities are involved. For instance, a child with limited upper-limb mobility should not be expected to operate a joystick. Ensuring accessibility is a fundamental precondition for playfulness, supporting enjoyment and engagement in the activity (Bundy et al., 2001).

### 5.3 Ethical considerations

In this section, we address ethical considerations in the context of play, an activity in which reality is intentionally suspended (Bundy et al., 2001). Within such contexts, a robot may need to exhibit emotional behaviors—either as part of its role or to engage the child, capture attention, stimulate interaction, or provide feedback. These behaviors are expected from an object showing animacy and the motivation for their presence is analogous to the one bringing children or caregivers to attribute traditional toys pretended emotional behaviors. Therefore we may consider them as ethically acceptable also in robot-mediated play. Furthermore, a robot in the play context can safely express, while maintaining a controlled, predictable behavior, emotions that a caregiver might not display-such as crying when “hit”-or that could be uncontrolled in a human playmate or pet. For certain children with social issues, experiencing or recognizing emotions constitutes a therapeutic objective. Within the play framework, also limited deception can be ethically admissible (de Oliveira et al., 2021; Zhang et al., 2019).

A related concern is that children may interact with robots instead of engaging with other humans. In the context of play, this situation is comparable to a child playing alone with a toy: interaction occurs, and new dimensions of play become available without supplanting human contact. Bonding with a robot may develop similarly to bonding with toys, video games, or other entertainment tools; interventions can be planned if needed. When other children or adults are present, the robot functions as an additional tool to design, enrich, or structure play activities. For children with disabilities, the caregiver’s role may shift from direct participation to supervision and facilitation, thereby fostering greater autonomy in the child’s development.

A final ethical consideration concerns the disclosure of sensitive data, such as dialogues between children and robots, over networks. Although such disclosure is prohibited without proper authorization, some toy manufacturers continue to embed natural language interaction in dolls and robots. This trend is also evident in social networks, video games, and large language models. Importantly, successful robot-mediated play does not necessarily require network connectivity; if network capabilities are used to support specific functionalities, they must fully comply with regulations such as the European GDPR.

## 6 Play activities

As already mentioned, the primary objective of any play activity is to immerse players in a state of “flow”, a psychological condition in which individuals are fully engaged, focused, and intrinsically motivated to continue the activity (Csikszentmihályi, 1997). Achieving this state requires to balance the difficulty of the task and of the interaction: the activity must not be too easy, which may lead to boredom, nor too difficult, which may result in frustration and early disengagement. Instead, the challenge should be calibrated to maintain interest and encourage sustained participation.

The way individuals engage in play is strongly influenced by the perceived affordances of both the robot and the play context. Elements such as the robot’s design, the physical environment, and any accompanying narrative contribute to shaping the player’s expectations and interaction patterns. These perceived affordances suggest possible actions and play dynamics. If a specific type of play is desired, both the robot and its context must be carefully designed to support and invite that form of engagement. However, as it is common, especially with children, players may deviate from the intended activity if they perceive a more appealing or stimulating alternative. In such cases, even a well-crafted narrative may be insufficient to address behavior within the planned boundaries of the activity, and the affordances of the robot or setting may instead lead to emergent, potentially even more playful interactions.

A critical element in play activity design is the formulation of goals, which may be implicit (emerging from the context) or explicitly stated. These goals must remain adaptable, as players can always reinterpret or modify them to better suit their interests, perceptions, or abilities. Such flexibility is essential for sustaining engagement and fostering meaningful, individualized play experiences.

In this section, we present examples of robots and play activities developed according to the proposed framework. These examples illustrate how the framework can guide design by highlighting which robot features and play characteristics are most relevant for achieving meaningful interactions and developmental outcomes. We include both successful implementations and cases revealing areas for improvement.

Some examples come from formally evaluated projects, with results summarized here and detailed in other papers. Others were only qualitatively assessed, as caregivers declined statistical analysis due to the diversity and specificity of participants, who cannot be assumed to belong to the same population. The limitations of statistical evaluation in this field are well recognized: Autism Spectrum Disorder is defined as a spectrum of many different states, sample sizes are typically small, and participant characteristics are difficult to capture (Scassellati et al., 2012; Schrum et al., 2020). Moreover, even in long-term interventions, performance may be influenced by factors external to the experience making its effects only partially observable (Aryania et al., 2020).

The aim of presenting these examples is to show how the framework offers a structured approach for designing and evaluating robot-assisted play activities, ensuring that key features of both robots and play are systematically considered. This repertoire of cases and experiences would shed a light on the possibilities of consciously implementing robots and play activities.

### 6.1 Practice play

**FIGURE 3:**
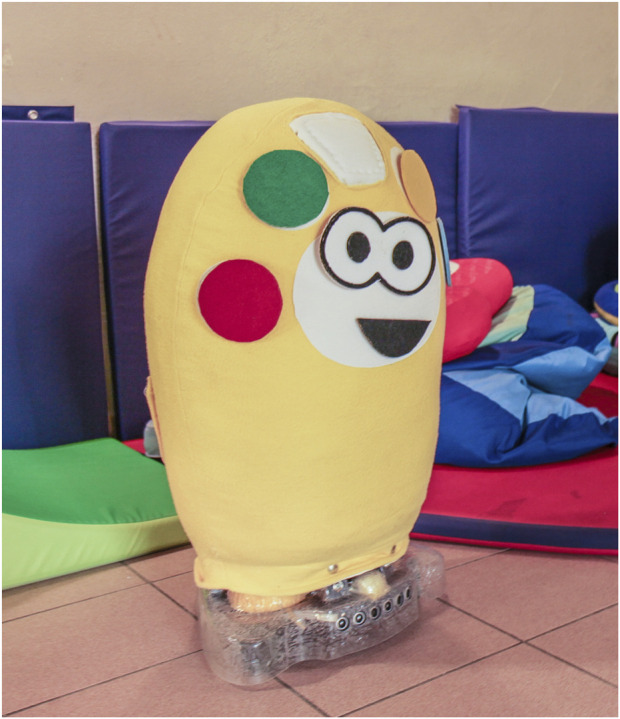
The robot Teo.

Teo (Bonarini et al., 2016; Brivio et al., 2021) is a soft, fabric-covered wheeled robot designed to support playful interaction (see Figure 3).

Its body allows for the attachment of Velcro™-mounted elements, enabling customization through the addition of features such as eyes or a mouth with specific expressions. This modularity fosters constructive play by encouraging children to actively manipulate and personalize the robot, while simultaneously supporting processes of emotional expression, symbolic representation, and gradual familiarization with the robotic agent. Teo is equipped with sensors for touch, distance, and mobility, allowing it to respond dynamically to user interaction, as well as with five touch-sensitive patches to enable direct, unambiguous semantic interaction. These patches have Velcro™ backing and can be attached to touch-sensitive areas implemented through capacitive sensors. Each patch can represent a play-relevant element, such as a color for matching tasks or an icon (e.g., a cow or a house) for question–answer or sound–association activities. Once a play activity is selected, Teo can employ the corresponding patches to support the specific goals of the game. In addition, Teo can be equipped with a repertoire of pre-recorded sounds, including spoken utterances, music for singing or dancing, and animal sounds for association tasks. A multicolored LED belt further enriches interaction by providing visual feedback through dynamic light cues.

Although Teo was initially designed to support rule-based games primarily centered on question-and-answer interactions, many noteworthy behaviors emerged during the familiarization phase—an unstructured period in which children were exposed to the robot without any specific instructions. In these spontaneous interactions, playful engagement often arose through simple action–reaction dynamics. In one such instance, a child with Autism Spectrum Disorder (ASD), upon noticing the robot’s movement, approached and forcefully pushed it over. In response, the robot emitted a crying sound and displayed slow, blue blinking lights to simulate sadness. The child, visibly surprised by the reaction, gently picked the robot up and then searched for a piece of cloth matching Teo’s color, which he offered to the robot as a gesture of reconciliation. This unprompted interaction highlights the potential of emotionally expressive robots to evoke empathy and foster social-emotional development in play contexts.

In another study (Bonarini and Besio, 2022), a girl with Down syndrome was invited to play a rule-based game with the robot Teo. However, the proposed activity did not initially capture her interest. After a period of hesitation, during which she remained approximately 1 m away from the robot, she noticed a piece of cloth on the floor. She used this cloth to initiate a form of interaction, showing it to Teo. The then remotely driven robot responded by tracking the cloth, prompting the girl to walk around while holding it, effectively leading the robot in a self-initiated and improvised game. This shift placed her in control of the interaction and engaged her in a novel, meaningful experience, which visibly increased her enjoyment. However, the interaction was disrupted when Teo was suddenly driven to move toward her too quickly, breaking her sense of safety and causing her to retreat—though she continued to observe the robot from a distance. Notably, when other children with neurodevelopmental disorders later entered the room, the same girl—who had previously shown no inclination to interact with peers—actively explained to them how to play with the robot. This example highlights the potential of robot play to foster agency, emotional engagement, and even social facilitation in children with developmental challengesas well as criticalities that may arise from wrong robot actions, in this case due to the decision, or the imperfect control, of the operator driving Teo. We would like to put in evidence that driving a playing robot requires skills that should be trained, but offers the possibility to exploit the robot features without the issues that autonomous behavior may rise. For a child with social problems, driving a robot may be a way to explore sociality from distance, for a care giver may be a way to act without a direct presence, which in some cases may trigger a rejection of a playful interaction.

**FIGURE 4:**
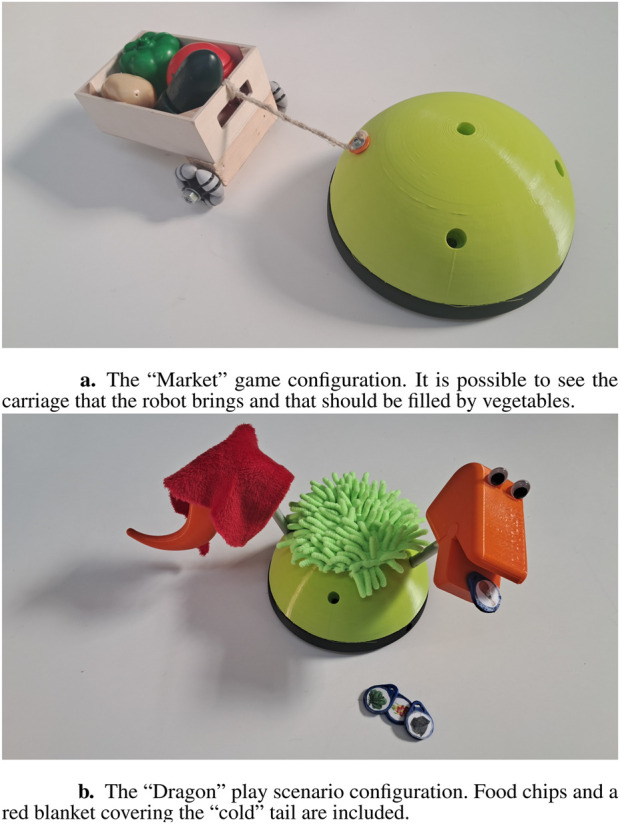
The robot FROBino. **(a)** The “Market” game configuration. It is possible to see the carriage that the robot brings and that should be filled by vegetables. **(b)** The “Dragon” play scenario configuration. Food chips and a red blanket covering the “cold” tail are included.

Teo has been tested in four different assistive associations, and one unit has been permanently adopted by one of them, where it has been used for more than 4 years with approximately 60 children presenting diverse neurodevelopmental disorders. Therapists employed Teo in both individual and small group sessions (3–5 children, aged 5 to 16 years), combining free play with structured activities. Thanks to the robot’s versatility, practitioners were able to adapt existing activities and easily design new ones. Observational reports highlighted several noteworthy outcomes: children with limited mobility followed the robot’s movements visually; children typically reluctant to engage in tactile interaction were motivated to hug or caress Teo in order to elicit its feedback; children usually sensitive to auditory stimuli tolerated and accepted the robot’s sounds within the play context; children with limited verbal communication expressed their desire to interact with the robot through gestures; and children with motor control difficulties demonstrated improved self-regulation in turn-taking. In some cases, initial sessions elicited heightened excitement, but this effect consistently diminished in subsequent encounters, suggesting that Teo contributed to the development of emotional regulation without requiring external intervention. For the FROB project, aimed at integration of children with disabilities in groups with typically developed children, we have implemented FROBino, a wheeled robot shaped like a dome, with a diameter of 20 cm (Figure 4). It is designed for use both on the floor and on tables, thanks to its sensors that prevent it from falling off edges. The robot is intended to be easily manipulated and interacted with. Its body is made of thermoplastic polyurethane (TPU), which provides resistance to accidental drops; however, its overall shape and dimensions are not intended to encourage dropping behaviors. On this main body of the robot several interchangeable modules can be attached to provide additional functionalities or aesthetic features.

The FROB project involved 27 classes from nursery and primary schools (ages 4–5 and 6–8, respectively), comprising approximately 350 children, 27 of whom had disabilities. The project aimed to evaluate whether robots could enhance the participation of children with disabilities in small-group play activities (4–5 participants) more effectively than equivalent traditional activities within the same play categories. Each class was visited twice, with sessions of approximately 90′ that included play activities drawn from the four basic categories, two at a time, both with robot and traditional toys.

Analysis of direct and video-recorded observations indicated that robot-mediated play promoted a greater variety and complexity of social interactions, particularly in primary school settings, compared to the corresponding traditional analogue activities. Cooperative play emerged in 44% of robot-based sessions and not with traditional toys, while transitions or overlaps between associative and cooperative play were observed in more than one-third of them. The introduction of robots stimulated a shift away from solitary or parallel play toward more complex, co-regulated forms of interaction.

In several cases, teachers played a crucial role in scaffolding the activities by adapting materials and roles to the diverse needs of participants. Moreover, children occasionally reconfigured the proposed activities autonomously, adapting them to the robot’s capabilities and to the players’ individual characteristics, thereby maintaining high levels of engagement and enjoyment.

“The Market” is an activity we developed for FROBino. Once the robot is powered on, it begins searching for objects to follow by rotating left and right. When FROBino detects an object directly in front of it and close enough, it starts moving toward the object. This behavior is facilitated by the use of a stick with a plastic carrot attached to the end, allowing children to guide FROBino around the room and direct it toward plastic vegetables scattered on the floor. The carrot serves as an intuitive and playful visual target, simplifying the task of maintaining the distance required to be followed by the robot. This approach is less demanding than having the children be directly followed by the robot, which requires precise control to match its pace and maintain the correct distance. When FROBino reaches a vegetable, children can retrieve the item and place it into the cart attached to the robot; this reinforces engagement through goal-oriented interaction.

The complexity of the interaction highlighted several important aspects. At the beginning of the experience, no explanation was given about the robot features, allowing the children to explore it freely. As a result, most children did not fully understand how to control the robot, assuming it would always follow the carrot regardless of its position. A few children who grasped the movement mechanics tried to explain it to their peers. This illustrates how the play activity can support the development of an understanding of complex behaviors and encourage the communication of such knowledge. However, while the children eventually developed their own ways of interacting with and controlling the robot, it is important to design the experience so that it can be adapted to the diverse needs and challenges children may present.

We were also able to reflect on the components used in the activity. Due to their wide availability, we employed plastic vegetables composed of two-halves held together by Velcro™, originally intended to pretend to “cut” them. However, we soon realized that these items became a source of distraction. Many children began to “multiply” the vegetables by separating the halves and spreading them around the room, while others created their own “mutant vegetables” by combining mismatched parts. Notably, some children with Autism Spectrum Disorders were particularly drawn to the sensory stimulation of throwing the vegetables into the air and watching them crash to the ground and fall apart with a loud noise. This highlights the importance of carefully selecting materials and designing activities in a way that minimizes potential distractions, especially when working with children with special needs.

### 6.2 Symbolic play

“The Dragon” is an activity we developed for FROBino (described in Section 6.1). During this activity, the robot moves around randomly and asks the children for help with various needs, such as when it “feels” hungry or cold. The children were expected to respond by assisting the robot, which in turn reacted to their actions by signaling whether its needs had been satisfied. For instance, if the robot appeared “hungry” and the children provided food by placing it in its mouth (detected through an RFID reader embedded in the robot that identified each tagged food item), it would respond with either an approving sound and movement (e.g., “Gnam, Gnam, Good!”) if the item was preferred, or with a gesture and sound of disgust if the item was undesired. This interaction not only introduced an element of surprise and emotional engagement but also supported the development of cognitive flexibility (by recognizing and adapting to different robot preferences), emotional understanding (through interpreting the robot’s affective reactions), and social regulation skills (particularly turn-taking and respecting shared resources). As the number of possible actions was limited and all children wished to participate, the activity provided natural opportunities to exercise patience, fairness, and turn-taking, occasionally requiring teacher facilitation to maintain balanced group dynamics.

A key aspect of the activity was the perception of the robot as a being with needs and preferences. This became especially evident when it expressed hunger: the children were presented with four types of food, three “good” options from which FROBino would randomly accept one, and one “bad” option that it would always reject. The robot’s consistent display of disgust toward the bad food was quickly understood. Two distinct behaviors emerged: some children ignored the bad food, recognizing that it did not help them achieve their goal of satisfying the robot, while others found its reaction amusing and repeatedly offered the bad option for fun.

Regarding the good food, some children experienced repeated rejections, purely due to random selection of the food they offered. While this often elicited laughter, in a few cases it led to frustration or anger, with children perceiving the robot as being unfair or deliberately uncooperative. These instances of negative reinforcement created opportunities for growth, as children either independently realized or were guided to understand that the robot, like other individuals, was not obligated to comply with every request. This helped reinforce the importance of respecting others in interactions.

### 6.3 Construction play

**FIGURE 5:**
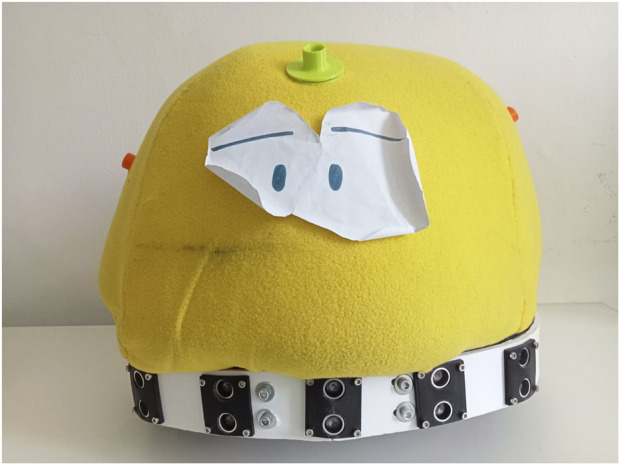
A version of the robot FROBone.

FROBone is a mobile dome approximately 40 cm tall with an oval shape, designed to be used on the ground (see Figure 5), within the FROB project. It mounts a belt of five sonar sensors on the front and two on the back to detect distances from objects. The body shape was designed to be as resistant and safe as possible during heavy interactions, while also allowing smooth, tactile, and visual engagement. FROBone’s identity is intended to be kind and gentle; for this reason, it has a rounded body shape and is covered with a soft fleece material. Due to these features, children’s initial reactions to the robot were consistently characterized by positive curiosity, and many hugged it. FROBone’s body includes slots for attaching modules that extend its functionalities. In addition, external interactive modules can be integrated into the play environment, further expanding the robot’s capabilities. By combining both the main body and the external modules, it is possible to implement several play scenarios. Each play scenario was guided by an adult supervisor who was responsible for introducing the activity.

In the play scenario “The Maze”, FROBone was programmed to avoid obstacles by moving straight when no barriers were present and turning right or left when impediments were detected on the sides. Once the children understood how it worked, the supervisor invited them to create a path using boxes and cones to guide the robot toward a visible, predefined target. This scenario was designed as a construction play activity, aimed at promoting coordination among the group in effectively placing obstacles.

In this play scenario, attention was captured by a short and sharp sound indicating the start of FROBone’s movement. The only engaging feature was the robot’s continuous motion, which would stop only near the target. The robot’s movement was governed by sonar-based distance measurements. However, factors such as numerous obstacles in the room, small play spaces and varying floor surfaces contributed to unclear and inconsistent robot behavior. This often led to a lack of understanding regarding the robot’s intentions. Participants’ memory was engaged by the need to recall the robot’s turning direction and the target location. The requirement to plan both the sequence and spatial arrangement of the obstacles supported participants’ planning abilities. Physical skills such as coordination and perception were tested through visual tracking of the robot movement and manual transport and placement of obstacles, which were made of lightweight fabric boxes. This play scenario required that all children in the group cooperate to achieve a common goal. The adult supervisor assigned turns to each child and ensured that they were respected. Decisions about where to place the boxes were made jointly and autonomously by the play group. The only type of communication emitted by the robot consisted of two sounds indicating the start and end of its movement.

Since some of the characteristics outlined in the framework where not met, in some cases problems arose. In the specific case of a girl with difficulties in verbal communication, manipulation, and a preference for solitary play, the given instructions and the robot’s movements were not clear enough for her to understand the intended goal. However, the collaboration activity intrinsically required by the context and the play activity encouraged her to verbally interact with the group throughout the entire session, maintaining steady attention on the ongoing activity. In other cases, the lack of clear instructions and auditory interactions, combined with small and distracting environments, made it difficult for some participants to stay focused on the activity. This resulted in frequent interventions from the supervisor and repeated clarifications of the play rules. These findings highlight the need to ensure engagement and clarity throughout the course of the activity. If the robot is not able to fulfill this task, the presence of a human supervisor is needed.

### 6.4 Rule-based play

**FIGURE 6:**
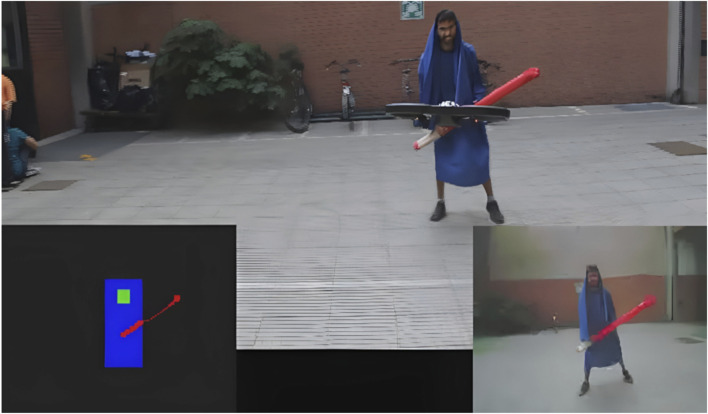
The Jedi Trainer game. On the bottom right the image from the onboard camera, on the left the its color-based interpretation.

We designed the Jedi Trainer game (Martinoia et al., 2013), where a drone was flying around a “Jedi trainee” holding a red pipe that represented a light saber, similarly to the Luke Skywalker training situation in the first movie of the Star Wars saga (see Figure 6). From time to time the drone was making a sound with the propellers similar to a “laser sound”. The player at that point had to bring the laser saber in front of the chest and the drone was able to detect whether the laser shot was parried. Key features to the success of this game were the clear signal of the significant event (laser shot sound), the ability of the drone to keep the distance, so to enforce perceived safety, and the “eye gaze” to the player (goal-direction), the score communicated to the player measuring successful and unsuccessful shots, and the strict time limit to play, which introduced a further dimension of the challenge.

Other two rule-based play scenarios were tested with FROBone, the robot already presented in Section 6.3.

**FIGURE 7:**
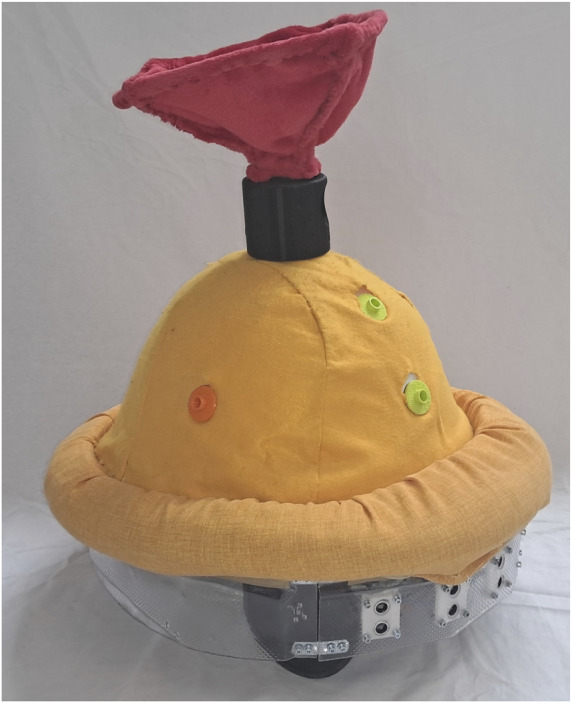
A version of the robot FROBone and the basket module.

In the play scenario “Basket”, a module was mounted on top of FROBone (a version of FROBone with basket is shown in Figure 7). The module consisted of a funnel covered with fleece fabric, placed on a cylinder with a lateral opening, featuring a 4 cm diameter hole matching the size of the balls used in the game. Each participant was given either a blue or a green ball. The goal of the game was to score by throwing the ball into the moving basket, as the robot wandered randomly around the room avoiding obstacles, while maintaining an alternating ball color sequence. At random intervals, FROBone would request a red ball to be thrown. Taking turns, the children had to retrieve the red ball and attempt to score. The game time was limited to 5 minutes.

Attention and understanding of the game were supported by audio cues emitted by the robot: before the activity began, the robot explained the game rules, and during the game, it provided feedback, confirming whether the color of the scored ball was correct. The presence of rules required both short-term and long-term memory. In this case, physical skills such as perception and coordination were stimulated through the need to listen for the color instructions (auditory perception) and to manipulate the balls, which were intentionally small and easily recognizable in color to enable also children with motion difficulties to play. Social cooperation skills were also supported by the color alternation rule and the one-ball-per-child distribution system, which encouraged turn-taking and cooperative behavior. The introduction of feedback on incorrect actions (throwing the wrong color in the sequence) had a deterrent effect on rule breaking. Many children, after hearing the robot sadly say “wrong”, paused and reflected on what to do next, often discussing their decisions with peers. In this case, the game was designed in alignment with the proposed framework. Improvements were observed in addition to a good level of sustained attention among all participants.

In particular, one child with significant difficulties in social interaction, maintaining attention, and group participation demonstrated positive outcomes by playing the basket game for a total of 15 min alongside four classmates, showing signs of distraction for approximately 2 minutes in total. His interest was captured from the moment FROBone explained the rules, and was maintained through the vocal feedback and the continuous motion of the robot’s wheels. Although his collaboration with peers was limited, he still engaged in the group activity. In another case, a girl with motion, attention, and interaction challenges was able to stay focused for the entire session. She participated actively, seeking support from her peers before and after each scoring attempt. Despite her manipulative difficulties, she had no significant problems handling the ball and throwing it independently.

**FIGURE 8:**
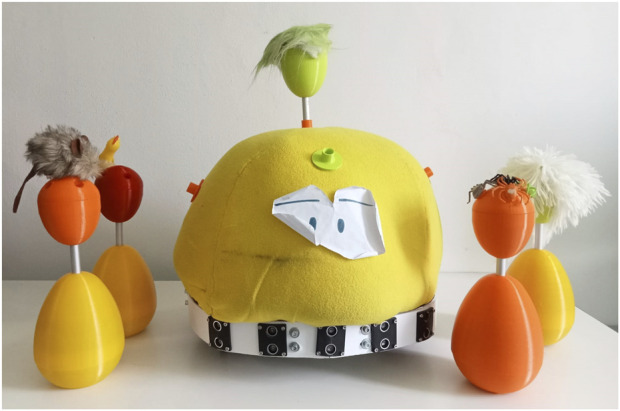
A version of the robot FROBone and modules used in the “Apprentice” play scenario.

Another rule-based play scenario is “The Apprentice”. It requires various types of modules in addition to the FROBone base (see Figure 8). This involved helping the robot, which interpreted an apprentice sorcerer, gather five ingredients to prepare a potion. In this case, the “ingredients” were egg-shaped modules (10 cm tall) made of soft material (3D-printed TPU), each holding an ingredient of the potion. At the beginning of the game, the ingredient eggs were scattered around the room, placed on pedestals made of larger egg-shaped modules (20 cm tall) made of the same material. The two modules (pedestal and ingredient) integrate an cheap ESP microprocessor that enables WI-FI connection and could be connected to each other and to the main body of FROBone using a removable rigid stick holding a CAN bus connection. The goal of the game was to guide FROBone to one pedestal at a time, in the order defined by the potion as asked by the robot. Children then had to retrieve the correct ingredient and attach it to the top of the robot using the rigid stick. Once all ingredients were collected and attached, the robot played a song and performed a short dance sequence.

This game supported attention and understanding through continuous feedback and explanations of the game rules, the insertion of each ingredient, and the successful completion of the potion. Negative feedback also helped focus the children’s attention on correct task execution, reinforcing rule-following behavior. Another key element that facilitated understanding was the precision of the robot’s movement: one child at a time, in turn, was required to guide FROBone to a specific point in the room by being followed (thanks to the sonar belt). The use of distance sensors and optimized wheel speed helped eliminate potential control difficulties. Memory was stimulated throughout the activity, as children had to remember the potion name and the correct order of its ingredients. To maintain a moderate difficulty level and ensure ongoing engagement, a “recipe book” was provided, listing four possible potions, each with its respective list of ingredients. Perceptual skills were engaged through multiple modalities: visual perception through the recipe book and ingredient localization; tactile perception through the soft materials of the modules; and auditory perception via the feedback and instructions provided by FROBone. Motion and coordination skills were also exercised during robot control and the transfer of ingredients from the pedestals to the robot’s mounting slots. Social skills, including turn-taking, role distribution, cooperation, and peer interaction, were continuously encouraged by the play dynamics. Often, during one participant’s turn, the rest of the group would cheer, give instructions, or suggest the correct ingredient, demonstrating teamwork. Finally, the entire group decided together which slot on FROBone should be used to place each ingredient. As in the previous scenario, this game was carefully aligned with the proposed framework. An increased attention level was observed among all participants and overall successful game completion.

In the specific case of a child with significant challenges in maintaining attention, cooperating with others, and following rules, this game proved to be effective. After receiving a negative feedback from the robot and understanding the rules, the child was able to guide FROBone accurately toward the correct ingredient, manipulate the module independently, insert it, and most importantly, cooperate with peers and wait for his turn without dominating the interaction.

## 7 Conclusion

The proposed framework for designing play activities involving robots effectively brings to the surface key elements of what is often an implicit, experience-based design process. The examples presented demonstrate that when the framework’s guidelines are followed, play activities tend to be successful, even with children facing physical, cognitive, or social challenges. Conversely, instances of reduced engagement or failure can often be analyzed and understood through the lens of the framework’s dimensions.

Future work will focus on extending the framework by integrating insights from new robot designs with diverse characteristics, as well as from a broader range of play scenarios. This iterative enrichment aims to further support inclusive, robot-based, and goal-oriented play activity design.

## Data Availability

The original contributions presented in the study are included in the article/supplementary material, further inquiries can be directed to the corresponding author.

## Footnotes

1
https://www.cost.eu/actions/TD1309/
